# Sperm DNA Fragmentation Is Associated with Impaired Directional Motility and Kinematic Efficiency: A CASA-Based Study

**DOI:** 10.3390/medicina62020376

**Published:** 2026-02-13

**Authors:** Ioana Cristina Rotar, Richard Buda, Adelin Marcu, Petronela Naghi, Liliana Sachelarie, David Călin Buzlea, Anca Huniadi, Mircea Ioan Sandor

**Affiliations:** 1 Department of Surgical Discipline, Faculty of Medicine and Pharmacy, UMF Iuliu Hațieganu, 410167 Cluj-Napoca, Romania; cristina.rotar@umfcluj.ro; 2 Calla-Infertility Diagnostic and Treatment Center, Constantin A. Rosetti Street, 410103 Oradea, Romania; buda.alexrichard@gmail.com (R.B.); adelinmarcu890@yahoo.com (A.M.); petronelanaghi@gmail.com (P.N.); ahuniadi@uoradea.ro (A.H.); 3 Doctoral School of Biological and Biomedical Sciences, Bihor County Emergency Clinical Hospital, University of Oradea, 410087 Oradea, Romania; 4 Preclinical Sciences Department, Faculty of Medicine, Apollonia University, 700511 Iasi, Romania; 5 Department of Preclinical Disciplines, Faculty of Medicine and Pharmacy, University of Oradea, 10 Piata 1 Decembrie Street, 410073 Oradea, Romania; 6 Department of Surgical Discipline, Faculty of Medicine and Pharmacy, University of Oradea, 1st December Square 10, 410073 Oradea, Romania; drims75@yahoo.com; 7 Pelican Clinical Hospital Oradea, Str. Corneliu Coposu nr. 14A-14B, 410450 Oradea, Romania; 8 CF Oradea Clinical Hospital, Str. Republic 56, 410159 Oradea, Romania

**Keywords:** sperm DNA fragmentation, computer-assisted semen analysis (CASA), sperm kinematic parameters, directional motility, male infertility

## Abstract

*Background and Objectives*: Sperm DNA fragmentation (SDF) has emerged as an important marker of male reproductive potential; however, its relationship with sperm kinematic performance remains incompletely understood. While conventional semen analysis primarily evaluates sperm concentration and motility, computer-assisted semen analysis (CASA) enables a more detailed assessment of sperm motility parameters, including velocity, path length, and directionality. *Materials and Methods*: This observational study included 183 semen samples, stratified by SDF levels into control (1–15%), mild (15.01–25%), moderate (25.01–50%), and severe (>50%) fragmentation groups. Sperm kinematic parameters were assessed using CASA, including curvilinear velocity (VCL), straight-line velocity (VSL), average path velocity (VAP), linearity (LIN), straightness (STR), and wobble (WOB). Group comparisons were performed using ANOVA or Kruskal–Wallis tests, correlation analyses were conducted using Spearman’s rank coefficient, and multivariable linear regression models were applied to evaluate the independent effect of SDF after adjustment for sperm concentration and progressive motility. *Results*: Parameters reflecting directional motility and movement efficiency differed significantly across increasing SDF categories. VSL, LIN, STR, and WOB showed a progressive decline with higher levels of DNA fragmentation, whereas VCL and VAP did not demonstrate a proportional decrease across groups. Correlation analysis revealed significant negative associations between SDF and VSL (ρ = −0.367, *p* < 0.001), VAP (ρ = −0.323, *p* < 0.001), and VCL (ρ = −0.202, *p* = 0.006), while correlations with LIN, STR, and WOB were negative but not statistically significant. Multivariable regression analysis confirmed SDF as an independent negative predictor of VSL and VAP after adjustment for conventional semen parameters, whereas the association with VCL was no longer significant. *Conclusions*: Increased sperm DNA fragmentation is associated with impaired sperm motility efficiency and directionality, rather than a uniform reduction in motility. These findings highlight the functional relevance of sperm DNA integrity in shaping sperm kinematic performance and support the complementary use of SDF assessment and CASA parameters in evaluating male fertility.

## 1. Introduction

Male infertility represents a complex and multifactorial condition, encompassing anatomical, physiological, hormonal, genetic, and molecular alterations that ultimately impair the fertilizing potential of spermatozoa. One of the earliest and most informative steps in the evaluation of male reproductive capacity is the assessment of sperm motility and kinematic behavior, as these parameters directly reflect the functional competence of sperm subpopulations within the ejaculate. Semen is inherently heterogeneous, comprising distinct sperm subpopulations characterized by varying degrees of progressive, non-progressive, or absent motility, conventionally classified as motility grades A, B, C, and D.

For a highly specialized cell such as the spermatozoon, fertilization capacity is critically dependent on both velocity and trajectory of movement. Advanced kinematic parameters, including curvilinear velocity (VCL), straight-line velocity (VSL), average path velocity (VAP), linearity index (LIN), straightness index (STR), and wobble (WOB), provide a detailed description of sperm movement patterns and their efficiency in navigating the female reproductive tract. These parameters, expressed in micrometers per second and percentage-based indices, offer a more refined evaluation of sperm functionality than conventional motility grading alone.

The introduction of computer-assisted semen analysis (CASA) systems has significantly enhanced the objective assessment of sperm kinematics, allowing precise quantification of movement patterns across different sperm subpopulations. Compared with traditional light microscopy, CASA provides superior reproducibility and sensitivity, enabling a more accurate interpretation of sperm physiological status and fertilization potential [1,2]. Nevertheless, data regarding the direct relationship between sperm velocity parameters and male fertility remain inconsistent, with greater emphasis traditionally placed on sperm concentration rather than motility dynamics.

In parallel, growing attention has focused on sperm DNA fragmentation (SDF) as a critical determinant of male fertility. SDF has been shown to adversely affect natural fertilization, embryo development, and assisted reproductive technology (ART) outcomes. Elevated SDF is primarily associated with defective chromatin maturation, abortive apoptosis, and oxidative stress (OS), and may be induced by various pathological conditions, including varicocele, inflammation, metabolic disorders, and adverse lifestyle or environmental exposures [3,4,5,6,7].

Oxidative stress arises when the generation of reactive oxygen species (ROS) exceeds the antioxidant capacity of seminal plasma. While physiological levels of ROS are essential for sperm maturation, capacitation, hyperactivation, acrosome reaction, and oocyte–sperm fusion, excessive ROS production leads to oxidative damage of nucleic acids, lipids, and proteins. Major sources of ROS include sperm transit through the seminiferous tubules and epididymis, as well as mitochondrial dysfunction, particularly in senescent spermatozoa [7,8]. Due to their limited cytoplasmic volume, sperm cells possess reduced antioxidant defenses, rendering their DNA particularly vulnerable to oxidative injury [9].

Excessive ROS induces the formation of oxidized DNA bases, such as 8-oxo-7,8-dihydro-2-deoxyguanosine (8-OHdG), which are excised by repair enzymes including 8-oxoguanine DNA glycosylase, resulting in unstable DNA structures highly susceptible to fragmentation [8,10]. When oxidative DNA lesions persist and are insufficiently repaired by the oocyte, they may give rise to de novo mutations or epimutations in the developing embryo, with potential transgenerational consequences [11].

Beyond DNA integrity, spermatozoa carry epigenetic information that influences offspring health. During spermatogenesis, sperm chromatin undergoes extensive remodeling, with histones largely replaced by protamines to ensure tight genomic compaction and transcriptional silencing. However, a small fraction of histones is retained at loci involved in early embryonic development and paternal epigenetic inheritance, which are regulated by histone methylation patterns, including H3K4 activation and H3K27 repression [12]. Abnormal protamination, defective protamine incorporation, or altered P1/P2 ratios have been associated with impaired fertilization and reduced pregnancy rates [11].

In addition, spermatozoa contain diverse RNA species that are highly susceptible to oxidative damage because they lack efficient repair mechanisms. Oxidative RNA modifications, driven by factors such as toxin exposure, inflammation, substance abuse, obesity, and poor nutrition, can reprogram sperm RNA profiles and have been linked to altered phenotypes in offspring. These findings support the concept of a multilayered epigenetic network through which oxidative stress may propagate its effects across generations [13].

Despite substantial evidence linking sperm DNA fragmentation to reproductive outcomes, the extent to which SDF alters sperm kinematic parameters and morphology within distinct sperm subpopulations remains incompletely understood. Clarifying whether DNA fragmentation directly impacts sperm velocity, trajectory, and morphokinetic behavior may provide new insights into the functional consequences of genomic instability and refine the interpretation of semen analysis in reproductive medicine.

The aim of this study was to determine whether increasing sperm DNA fragmentation is associated with impaired sperm kinematic performance, with particular emphasis on directional motility and movement efficiency rather than absolute velocity alone. Using computer-assisted semen analysis (CASA), we evaluated key kinematic parameters to determine whether sperm DNA fragmentation indicates functional deficits in sperm motility that may remain undetected by conventional semen analysis, despite apparently normal sperm concentration and progressive motility.

While previous studies have reported associations between sperm DNA fragmentation and conventional motility parameters, the present study specifically focuses on CASA-derived indices of directional motility and movement efficiency. By integrating clinically relevant sperm DNA fragmentation thresholds with detailed kinematic analysis and multivariable adjustment, this work aims to clarify whether sperm DNA fragmentation primarily affects movement quality rather than absolute sperm velocity.

## 2. Materials and Methods

### 2.1. Study Design and Sample Population

This observational study included 183 semen samples obtained from men undergoing fertility evaluation. All samples were collected after a period of sexual abstinence in accordance with standard laboratory practice and processed following complete liquefaction. Semen analysis was performed under standardized laboratory conditions, in compliance with the World Health Organization (WHO) Laboratory Manual for the Examination and Processing of Human Semen, 6th edition. Ethical approval for the retrospective analysis of thesecollected data was granted by the Institutional Review Board of Calla IVF Center (No. 1123/A/22 February 2024). Each semen sample analyzed in this study corresponded to a unique participant. Basic clinical and semen-related variables with potential influence on sperm DNA fragmentation and motility, including abstinence period and conventional semen parameters, were recorded at the time of sample collection.

### 2.2. Assessment of Sperm DNA Fragmentation and Group Stratification

Sperm DNA fragmentation (SDF) was assessed using a sperm chromatin dispersion (SCD)–based assay (halo test), routinely used in clinical laboratory practice. Following complete liquefaction, semen samples were gently homogenized, and a 200 µL aliquot from each sample was transferred into a 1.5 mL Eppendorf tube. A volume of 100 µL of sperm wash medium (Fertipro, Beernem, Belgium) was carefully layered over the sample, and a swim-up procedure was performed. Samples were incubated for 1 h to allow motile spermatozoa to migrate into the upper fraction. After incubation, 20 µL of the supernatant containing motile spermatozoa was collected and mixed with 40 µL of melted agarose. From the resulting mixture, 8 µL was placed onto a specially treated microscope slide and covered with a coverslip. Slides were refrigerated for 5 min to allow the agarose to solidify. Subsequently, slides were treated sequentially with a denaturation solution for 7 min and a lysis solution for 20 min. After lysis, slides were rinsed with distilled water for 5 min, dehydrated in 70% ethanol for 2 min followed by 100% ethanol for an additional 2 min, and then air-dried. Differential staining was performed with eosin for 7 min, followed by thiazine for 7 min.

Prepared slides were examined under light microscopy with a 10× objective using a dedicated image-capture system for DNA fragmentation analysis. Spermatozoa were classified based on halo morphology as spermatozoa without DNA fragmentation, characterized by large halos equal to or greater than the diameter of the sperm head, and spermatozoa with DNA fragmentation, characterized by small halos (approximately one-third of the head diameter), absence of halos, or highly degraded nuclei. The percentage of spermatozoa exhibiting DNA fragmentation was calculated for each sample.

Based on the proportion of spermatozoa with fragmented DNA, samples were stratified into four groups reflecting increasing levels of genomic damage: Group 1—Control (Normozoospermia) (n = 22), defined by SDF values of 1–15%, sperm concentration >16 million/mL, progressive motility > 30%, and teratozoospermia indices within World Health Organization (WHO) reference limits (TZI < 2.07, MAI < 2.38, SDI < 2.3); Group 2—Mild DNA fragmentation (n = 99), SDF 15.01–25%; Group 3—Moderate DNA fragmentation (n = 53), SDF 25.01–50%; and Group 4—Severe DNA fragmentation (n = 9), SDF 51.01–70%.

This stratification enabled evaluation of progressive alterations in sperm kinematic performance with increasing sperm DNA fragmentation.

### 2.3. Computer-Assisted Semen Analysis (CASA)

Sperm motility and kinematic characteristics were evaluated using computer-assisted semen analysis (CASA), which provides an objective and reproducible assessment of sperm movement patterns and functional performance. CASA allows detailed analysis of sperm velocity, trajectory, and movement efficiency across different sperm subpopulations.

After complete liquefaction, semen samples were gently homogenized using a sterile Pasteur pipette. A 20 µL aliquot of each sample was loaded into a Leja counting chamber and analyzed using a computer-assisted semen analysis (CASA) system. Sperm motility analysis was performed using a Nikon Eclipse Ci microscope (Nikon Instruments Inc., Tokyo, Japan) equipped with CASA software specifically designed for motility assessment (SCA® (Sperm Class Analyzer) (Microptic S.L., Barcelona, Spain), software version 6.4.0.1). For each sample, a minimum of 500 spermatozoa were recorded and automatically analyzed. The system provided objective measurements of sperm concentration, progressive motility, total motility, and immotility, as well as mean kinematic parameters, including curvilinear velocity (VCL), straight-line velocity (VSL), average path velocity (VAP), linearity index (LIN), straightness index (STR), and wobble (WOB). All analyses were conducted under standardized laboratory conditions to ensure reproducibility and consistency.

The following kinematic parameters were analyzed: Curvilinear velocity (VCL): velocity along the actual curvilinear trajectory (µm/s); Straight-line velocity (VSL): velocity along a straight line from the starting point to the endpoint (µm/s); Average path velocity (VAP): velocity along a smoothed average trajectory (µm/s); Linearity (LIN): calculated as VSL/VCL × 100 (%); Straightness (STR): calculated as VSL/VAP × 100 (%); Wobble (WOB): calculated as VAP/VCL × 100 (%), reflecting lateral head displacement and oscillatory movement. These parameters provide a comprehensive functional characterization of sperm motility, distinguishing absolute velocity from directional efficiency and movement stability.

### 2.4. Conventional Semen Parameters and Morphological Indices

In addition to CASA-derived parameters, conventional semen characteristics were recorded for all samples, including sperm concentration and progressive motility (grades A + B). Sperm morphology was assessed using standardized indices of teratozoospermia, namely the Teratozoospermia Index (TZI), Multiple Anomalies Index (MAI), and Sperm Deformity Index (SDI), following WHO recommendations. These parameters were used to characterize the study population and for exploratory analyses but were not considered primary functional endpoints.

### 2.5. Outcome Measures

The primary outcomes of the study were CASA-derived kinematic parameters reflecting directional motility and movement efficiency, including VSL, LIN, STR, and WOB. Secondary descriptive parameters included VCL and VAP, as well as conventional semen parameters used for population characterization.

### 2.6. Statistical Analysis

Statistical analyses were performed using IBM SPSS Statistics version 26.0 (IBM Corp., Armonk, NY, USA). Continuous variables were assessed for normality using the Shapiro–Wilk test. Normally distributed data are presented as mean ± standard deviation, whereas non-normally distributed variables are reported as median with interquartile range (IQR). Comparisons of sperm kinematic parameters and conventional semen characteristics across the four sperm DNA fragmentation (SDF) categories were conducted using one-way analysis of variance (ANOVA) for normally distributed variables and the Kruskal–Wallis test for non-normally distributed variables, as appropriate. Post hoc pairwise comparisons were performed with multiple-testing adjustment. Given the unequal group sizes, particular emphasis was placed on the consistency and direction of effects observed across increasing SDF categories. Primary outcome measures included CASA-derived parameters reflecting directional motility and movement efficiency (straight-line velocity [VSL], linearity [LIN], straightness [STR], and wobble [WOB]), while curvilinear velocity (VCL) and average path velocity (VAP) were considered secondary descriptive kinematic variables.

Associations between sperm DNA fragmentation, analyzed as a continuous variable, and CASA-derived kinematic parameters were evaluated using Pearson or Spearman correlation coefficients, depending on data distribution. To assess whether sperm DNA fragmentation independently influenced sperm kinematic performance, multivariable linear regression models were constructed, with selected CASA-derived parameters as dependent variables and SDF as the main predictor, adjusting for conventional semen parameters, including sperm concentration and progressive motility. All statistical tests were two-tailed, and a *p*-value < 0.05 was considered statistically significant.

The primary purpose of the multivariable regression analyses was to assess the independence of associations between sperm DNA fragmentation and kinematic parameters after adjustment for conventional semen variables, rather than to maximize explained variance or predictive performance.

## 3. Results

**Figure 1:**
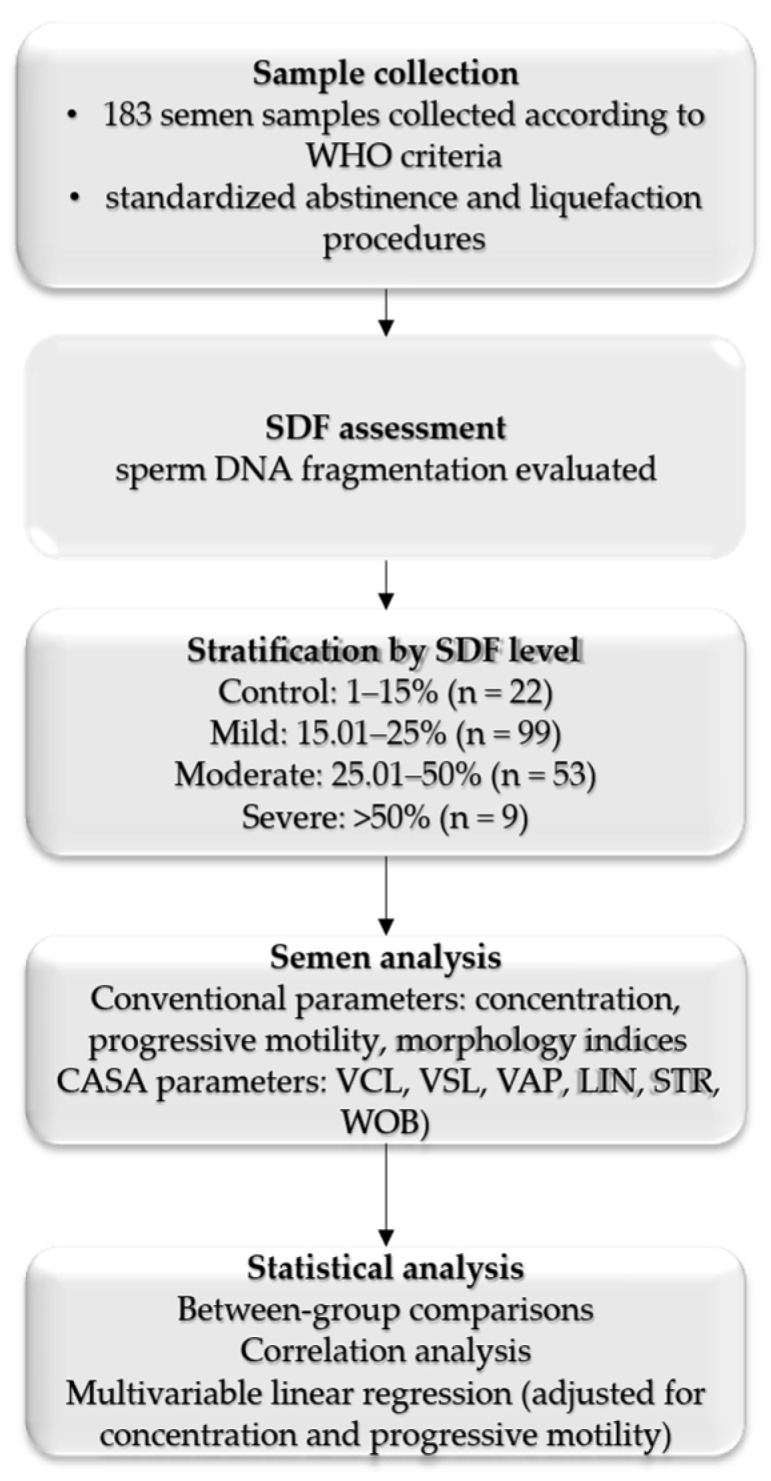
Study workflow.

The overall study design and analytical workflow are summarized in Figure 1.

### 3.1. Characteristics of the Study Population

A total of 183 semen samples were included in the analysis and stratified into four groups based on sperm DNA fragmentation (SDF) levels. The control group (SDF 15%) comprised 22 samples fulfilling World Health Organization (WHO) criteria for normozoospermia, including normal sperm concentration, progressive motility, and morphological indices within reference limits. The remaining samples were classified as mild (SDF 25%, n = 99), moderate (SDF 50%, n = 53), or severe (SDF –70%, n = 9) DNA fragmentation.

Sperm concentration varied across study groups but remained within clinically relevant ranges in all categories. Progressive motility (grades A + B) was preserved across groups, although a tendency toward lower values was observed in samples with higher SDF levels. Morphological assessment, as measured by the teratozoospermia index (TZI), sperm deformity index (SDI), and multiple anomalies index (MAI), showed increasing variability with increasing DNA fragmentation, while remaining within predefined descriptive ranges for each group.

Overall, the stratification enabled the comparison of sperm kinematic performance across increasing levels of DNA fragmentation in a population characterized by heterogeneous but clinically representative semen parameters.

Baseline semen characteristics and morphological indices of the study population, stratified by sperm DNA fragmentation categories, are presented in Table 1.

### 3.2. Comparison of CASA Kinematic Parameters Across SDF Groups

Comparisons of CASA-derived sperm kinematic parameters across the four sperm DNA fragmentation (SDF) groups demonstrated statistically significant differences for parameters reflecting directional motility and movement efficiency.

Straight-line velocity (VSL) showed significant differences across SDF categories (*p* < 0.05), whereas linearity-related indices (LIN, STR, and WOB) exhibited decreasing trends that did not consistently reach statistical significance across groups. Post hoc analyses revealed progressively lower values of these parameters in the moderate and severe SDF groups compared with the control group, indicating a gradual impairment of directional sperm movement with increasing DNA fragmentation.

In contrast, absolute velocity parameters, including curvilinear velocity (VCL) and average path velocity (VAP), did not show a consistent statistically significant decrease across SDF groups. Although intergroup variability was observed, no proportional trend in DNA fragmentation was identified for these parameters (*p* > 0.05 for trend).

Overall, statistical comparisons across SDF categories demonstrated that sperm DNA fragmentation is primarily associated with alterations in directional and efficiency-related kinematic parameters, while measures of overall movement speed were relatively preserved.

Spermatozoa remain motile; however, their movement becomes less directionally efficient rather than uniformly slower. CASA-derived sperm kinematic parameters across increasing sperm DNA fragmentation categories are summarized in Table 2.

### 3.3. Correlation Between SDF and Directional Motility Parameters

Correlation analysis revealed significant associations between sperm DNA fragmentation (SDF), analyzed as a continuous variable, and CASA-derived parameters reflecting directional motility and movement efficiency.

Sperm DNA fragmentation showed a significant negative correlation with straight-line velocity (VSL), whereas correlations with linearity-related indices (LIN, STR, and WOB) were negative but did not reach statistical significance, indicating that increasing DNA fragmentation primarily affects forward progression rather than overall trajectory linearity. These correlations were moderate in strength and consistent across the analyzed population.

In contrast, correlations between SDF and parameters reflecting absolute velocity, including curvilinear velocity (VCL) and average path velocity (VAP), were weak and not statistically significant (*p* > 0.05), highlighting the limited sensitivity of these measures to increasing DNA fragmentation.

Overall, correlation analysis demonstrated that sperm DNA fragmentation is more strongly associated with alterations in directional and efficiency-related kinematic parameters than with overall movement speed.

As shown in Table 3, correlation analysis indicated that increasing sperm DNA fragmentation was significantly associated with reduced sperm kinematic performance. Straight-line velocity (VSL) exhibited a moderate and statistically significant negative correlation with SDF (ρ = −0.367, *p* < 0.001), while average path velocity (VAP) was also negatively correlated with SDF (ρ = −0.323, *p* < 0.001), indicating impaired efficiency of sperm movement with increasing DNA fragmentation. Curvilinear velocity (VCL) showed a weaker but significant negative association with SDF (ρ = −0.202, *p* = 0.006), suggesting that overall movement speed was less affected than directional progression. In contrast, indices of movement linearity and stability, including LIN, STR, and WOB, demonstrated negative but non-significant correlations (*p* > 0.05), indicating a trend toward reduced directional control that did not follow a strong linear relationship across the entire study population.

Despite preserved motility, increased sperm DNA fragmentation is associated with impaired directional control and reduced sperm motility efficiency.

Correlations were assessed using Spearman’s rank correlation analysis. A *p*-value < 0.05 was considered statistically significant. While VSL and VAP showed robust negative associations with sperm DNA fragmentation, the correlation with VCL was weaker, and indices of movement linearity and stability did not demonstrate significant linear relationships.

### 3.4. Multivariable Analysis

To evaluate whether sperm DNA fragmentation independently influenced sperm kinematic performance, multivariable linear regression analyses were performed with selected CASA-derived parameters as dependent variables. Straight-line velocity (VSL), average path velocity (VAP), and curvilinear velocity (VCL) were included as outcome variables, while sperm DNA fragmentation was entered as the main predictor. Models were adjusted for conventional semen parameters, including sperm concentration and progressive motility.

After adjustment for these confounding factors, sperm DNA fragmentation remained a significant independent negative predictor of straight-line velocity (VSL) and average path velocity (VAP) (*p* < 0.05), indicating that increasing levels of DNA fragmentation were associated with impaired sperm motility efficiency, irrespective of sperm concentration and overall motility status. The association between SDF and curvilinear velocity (VCL) was weaker and did not retain consistent statistical significance after multivariable adjustment.

Overall, multivariable analysis confirmed that the relationship between sperm DNA fragmentation and impaired sperm kinematic performance was not solely explained by conventional semen parameters, supporting an independent association between genomic integrity and sperm movement efficiency. Multivariable regression analysis confirmed sperm DNA fragmentation as an independent negative predictor of directional sperm movement parameters, even after adjustment for conventional semen characteristics (Table 4).

Multivariable analysis confirmed sperm DNA fragmentation as an independent negative predictor of straight-line and average path velocities, even after adjustment for sperm concentration and progressive motility. In contrast, curvilinear velocity was not independently associated with SDF, indicating that DNA fragmentation primarily affects the efficiency and directionality of sperm movement rather than overall motility.

## 4. Discussion

The present study provides a detailed analysis of the relationship between sperm DNA fragmentation (SDF) and sperm kinematic performance, as assessed by computer-assisted semen analysis (CASA), with particular emphasis on parameters reflecting movement efficiency and directional control. While conventional semen analysis primarily focuses on sperm concentration and motility, CASA enables a more refined functional evaluation of sperm movement, which is increasingly recognized as relevant for fertility outcomes [1,2].

Importantly, our findings extend existing CASA–SDF literature by demonstrating a functional dissociation between preserved sperm motility and impaired directional efficiency, indicating that sperm DNA fragmentation predominantly compromises movement quality rather than inducing a uniform reduction in sperm speed.

Our findings demonstrate that increasing sperm DNA fragmentation is associated with progressive alterations in sperm motility patterns, predominantly affecting straight-line velocity (VSL) and trajectory-efficiency parameters. These results are consistent with previous studies reporting that sperm motility is a multidimensional trait and that directional movement plays a crucial role in fertilization competence [1,14,15,16]. Spermatozoa must not only remain motile but also sustain a directed and efficient trajectory to successfully navigate the female reproductive tract.

Notably, absolute velocity parameters such as curvilinear velocity (VCL) and average path velocity (VAP) did not decrease in a proportional manner across SDF groups. This suggests that spermatozoa with increased DNA fragmentation may retain some motility yet exhibit reduced efficiency in translating motion into forward progression. Similar discrepancies between overall velocity and movement quality have been described in CASA-based studies analyzing sperm subpopulations in both human and animal models [2,15,16]. These observations reinforce the concept that sperm speed alone is insufficient to characterize functional motility.

Correlation analysis further supported this interpretation, revealing significant negative associations between SDF and VSL, VAP, and VCL, while correlations with linearity-related indices were weaker and did not reach statistical significance across the entire cohort. This pattern likely reflects the biological heterogeneity of ejaculates, in which sperm subpopulations with distinct motility profiles and DNA integrity levels coexist [15,16]. Consequently, global correlation analyses may underestimate the impact of DNA fragmentation on specific functional sperm subgroups.

Importantly, multivariable regression analysis confirmed sperm DNA fragmentation as an independent negative predictor of straight-line and average path velocities after adjustment for conventional semen parameters, including sperm concentration and progressive motility. This finding indicates that the observed alterations in sperm kinematics cannot be explained solely by reductions in sperm count or general motility. Instead, SDF appears to represent a distinct functional dimension of sperm quality that independently influences motility efficiency [3,4,5,10,17].

Several biological mechanisms may underlie the association between increased DNA fragmentation and reduced sperm motility [18,19,20,21,22]. Oxidative stress is widely recognized as a central contributor to sperm DNA damage and has been shown to negatively affect mitochondrial function, axonemal integrity, and membrane fluidity, all of which are essential for coordinated flagellar motion [6,7,8,9,23]. Moreover, defective chromatin packaging and altered protamination may modify sperm head biomechanics, potentially impairing hydrodynamic behavior and directional stability during movement through viscous environments such as cervical mucus [11,12,24].

Beyond oxidative damage, emerging evidence suggests that epigenetic alterations and transcriptomic dysregulation in spermatozoa may reflect broader disturbances in spermatogenesis that simultaneously affect genomic integrity and motility-related functions [12,13,25]. These molecular changes may contribute to reduced functional competence of spermatozoa with elevated DNA fragmentation, even when motility is preserved.

From a clinical perspective, these findings highlight the limitations of conventional semen analysis in identifying subtle yet functionally relevant sperm defects. Samples classified as normozoospermic based on concentration and motility may still harbor spermatozoa with impaired directional movement associated with increased DNA fragmentation [26]. This observation aligns with previous reports linking high SDF levels to reduced natural conception rates, impaired assisted reproductive technology outcomes, and increased risk of early embryonic loss [3,4,10,17,27,28].

The integration of SDF testing with CASA-derived kinematic analysis may therefore provide a more comprehensive functional assessment of male fertility potential. Such an approach could be particularly valuable in cases of unexplained infertility or discordant clinical findings, where standard semen parameters fail to fully explain reproductive failure. Advanced motility analyses have already demonstrated predictive value in veterinary and experimental settings, supporting their translational relevance in human reproductive medicine [14,15,16,29,30].

Several limitations of this study should be acknowledged. The observational design precludes causal inference, and the unequal distribution of samples across SDF categories, particularly in the severe fragmentation group, may limit statistical power for certain comparisons. Additionally, molecular markers of oxidative stress and mitochondrial function were not directly assessed. Future studies integrating biochemical, molecular, and subpopulation-based analyses of motility may provide further insight into the mechanisms linking sperm DNA fragmentation to altered kinematic performance.

An important limitation of this study is the unequal distribution of samples across sperm DNA fragmentation categories, particularly the small number of cases in the severe fragmentation group. This imbalance may have limited statistical power for group-wise comparisons and reduced the precision of estimates specific to this category. To mitigate this effect, sperm DNA fragmentation was also analyzed as a continuous variable, and multivariable regression models were applied across the entire cohort. Accordingly, findings related to severe DNA fragmentation should be interpreted with caution and considered exploratory.

Sperm DNA fragmentation was assessed using the sperm chromatin dispersion (SCD) assay, which reflects susceptibility of sperm chromatin to denaturation rather than directly quantifying the number or type of DNA strand breaks. While SCD is a robust, clinically practical method, it does not distinguish between single- and double-strand breaks. Other techniques, such as TUNEL or Comet assays, provide complementary information but involve greater technical complexity, whereas SCSA requires specialized flow cytometry equipment. Therefore, the present findings should be interpreted within the methodological framework of the SCD assay.

Another limitation is the absence of reproductive outcome data, including natural conception rates or assisted reproductive technology outcomes. Consequently, the observed associations cannot be directly translated into fertility prognosis or treatment success. In addition, the observational study design precludes causal inference, and motility assessment was performed under in vitro conditions that may not fully reflect the dynamic environment of the female reproductive tract. Finally, molecular markers of oxidative stress, mitochondrial function, and chromatin organization, as well as sperm subpopulation analyses, were not included, limiting mechanistic interpretation.

This study demonstrates that increased sperm DNA fragmentation is associated with impaired sperm motility efficiency and directionality, independently of conventional semen parameters. These findings support the functional relevance of sperm DNA integrity in shaping sperm kinematic behavior and highlight the value of integrating sperm DNA fragmentation assessment with CASA-based kinematic analysis in the evaluation of male fertility. While this combined approach may provide valuable functional insight, particularly in cases of unexplained male infertility, its direct clinical utility requires validation in future studies incorporating reproductive outcome measures and longitudinal designs.

## 5. Conclusions

This study demonstrates that increased sperm DNA fragmentation is associated with impaired sperm kinematic efficiency, particularly affecting directional movement parameters rather than overall motility. While spermatozoa may retain motility, elevated DNA fragmentation compromises their ability to maintain effective forward progression. Importantly, sperm DNA fragmentation emerged as an independent predictor of altered kinematic performance after adjustment for conventional semen parameters.

These findings underscore the functional relevance of sperm DNA integrity in shaping sperm movement quality and support the complementary use of sperm DNA fragmentation testing and CASA-based kinematic analysis as part of an expanded functional assessment of male fertility. However, in the absence of reproductive outcome data, the clinical implications of these associations should be interpreted with caution. Further studies integrating molecular, kinematic, and reproductive outcome measures are warranted to clarify the translational and clinical applicability of these combined biomarkers.

## Figures and Tables

**Table 1:** Baseline characteristics of the study population stratified by sperm DNA fragmentation (SDF).

Parameter	Control (SDF 1–15%) (n = 22)	Mild SDF (15.01–25%) (n = 99)	Moderate SDF (25.01–50%) (n = 53)	Severe SDF (51.01–70%) (n = 9)
Sperm concentration (million/mL)	36.9 ± 23.4	28.1 ± 26.6	18.1 ± 19.5	23.3 ± 19.5
Progressive motility (A + B, %)	53.2 ± 18.5	47.8 ± 21.0	40.1 ± 22.3	25.2 ± 9.8
Teratozoospermia Index (TZI)	1.29 ± 0.11	—	—	—
Sperm Deformity Index (SDI)	—	2.80 ± 0.47	2.64 ± 0.53	2.72 ± 0.65
Multiple Anomalies Index (MAI)	2.11 ± 0.20	—	—	—

Note: Data are presented as mean ± standard deviation. This table summarizes baseline semen characteristics and morphological indices used for population description. No inferential statistical comparisons were performed for baseline characteristics.

**Table 2:** Comparison of CASA-derived kinematic parameters across sperm DNA fragmentation (SDF) groups.

Parameter	Control (SDF 1–15%) (n = 22)	Mild SDF (15.01–25%) (n = 99)	Moderate SDF (25.01–50%) (n = 53)	Severe SDF (>50%) (n = 9)
VCL (µm/s)	42.61 ± 9.35	39.98 ± 11.15	38.21 ± 10.68	31.59 ± 10.10
VSL (µm/s)	20.74 ± 4.40	19.28 ± 5.82	16.28 ± 5.47	13.81 ± 6.34
VAP (µm/s)	28.65 ± 5.00	25.90 ± 6.06	23.55 ± 5.80	20.08 ± 8.38
LIN (%)	49.77 ± 10.20	49.81 ± 8.71	47.04 ± 14.07	43.45 ± 18.23
STR (%)	69.80 ± 7.58	71.00 ± 6.67	67.49 ± 12.27	70.41 ± 15.55
WOB (%)	68.85 ± 7.48	67.51 ± 6.95	65.47 ± 10.86	62.08 ± 21.47

Note: Data are presented as mean ± standard deviation. Comparisons between groups were performed using one-way ANOVA or Kruskal–Wallis test, as appropriate.

**Table 3:** Correlation between sperm DNA fragmentation and CASA-derived kinematic parameters.

Parameter	Spearman ρ	*p*-Value	n
VCL	−0.202	0.006087	183
VSL	−0.367	3.148 × 10^−7^	183
VAP	−0.323	8.109 × 10^−6^	183
LIN	−0.131	0.07602	183
STR	−0.124	0.09348	183
WOB	−0.100	0.1795	183

**Table 4:** Multivariable linear regression analysis of CASA-derived kinematic parameters.

Outcome	Predictor	β Coefficient	Std. Error	*p*-Value	95% CI
VSL	SDF	−0.315	0.155	0.045	[−0.622, −0.008]
VSL	Concentration	−0.004	0.016	0.798	[−0.036, 0.027]
VSL	Progressive motility	0.155	0.020	0.000	[0.115, 0.196]
VAP	SDF	−0.283	0.142	0.048	[−0.564, −0.002]
VAP	Concentration	−0.003	0.015	0.858	[−0.031, 0.026]
VAP	Progressive motility	0.201	0.019	0.000	[0.164, 0.238]
VCL	SDF	−0.358	0.255	0.163	[−0.863, 0.147]
VCL	Concentration	−0.058	0.026	0.029	[−0.110, −0.006]
VCL	Progressive motility	0.386	0.034	0.000	[0.319, 0.452]

Models were adjusted for sperm concentration and progressive motility. β coefficients represent the change in the outcome variable per unit increase in the predictor.

## Data Availability

The original contributions presented in this study are included in the article. For further inquiries, please contact the corresponding authors.
